# Racial, Ethnic, and Language-Based Inequities in Inpatient Opioid Prescribing by Diagnosis from Internal Medicine Services, a Retrospective Cohort Study

**DOI:** 10.1155/2023/1658413

**Published:** 2023-09-21

**Authors:** Mihir Joshi, Priya A. Prasad, Colin C. Hubbard, Nicholas Iverson, Solmaz P. Manuel, Margaret C. Fang, Aksharananda Rambachan

**Affiliations:** ^1^Santa Clara Valley Medical Center, San Jose, USA; ^2^University of California San Francisco, Department of Medicine, Division of Hospital Medicine, San Francisco, USA; ^3^Division of Hospital Medicine, San Francisco General Hospital and Trauma Center, San Francisco, California, USA; ^4^University of California San Francisco, Department of Anesthesia and Perioperative Care, San Francisco, USA

## Abstract

**Introduction:**

Opioid administration is extremely common in the inpatient setting, yet we do not know how the administration of opioids varies across different medical conditions and patient characteristics on internal medicine services. Our goal was to assess racial, ethnic, and language-based inequities in opioid prescribing practices for patients admitted to internal medicine services.

**Methods:**

We conducted a retrospective cohort study of all adult patients admitted to internal medicine services from 2013 to 2021 and identified subcohorts of patients treated for the six most frequent primary hospital conditions (pneumonia, sepsis, cellulitis, gastrointestinal bleed, pyelonephritis/urinary tract infection, and respiratory disease) and three select conditions typically associated with pain (abdominal pain, acute back pain, and pancreatitis). We conducted a negative binomial regression analysis to determine how average administered daily opioids, measured as morphine milligram equivalents (MMEs), were associated with race, ethnicity, and language, while adjusting for additional patient demographics, hospitalization characteristics, medical comorbidities, prior opioid therapy, and substance use disorders.

**Results:**

The study cohort included 61,831 patient hospitalizations. In adjusted models, we found that patients with limited English proficiency received significantly fewer opioids (66 MMEs, 95% CI: 52, 80) compared to English-speaking patients (101 MMEs, 95% CI: 91, 111). Asian (59 MMEs, 95% CI: 51, 66), Latinx (89 MMEs, 95% CI: 79, 100), and multi-race/ethnicity patients (81 MMEs, 95% CI: 65, 97) received significantly fewer opioids compared to white patients (103 MMEs, 95% CI: 94, 112). American Indian/Alaska Native (227 MMEs, 95% CI: 110, 344) patients received significantly more opioids. Significant inequities were also identified across race, ethnicity, and language groups when analyses were conducted within the subcohorts. Most notably, Asian and Latinx patients received significantly fewer MMEs and American Indian/Alaska Native patients received significantly more MMEs compared to white patients for the top six most frequent conditions. Most patients from minority groups also received fewer MMEs compared to white patients for three select pain conditions. *Discussion*. There are notable inequities in opioid prescribing based on patient race, ethnicity, and language status for those admitted to inpatient internal medicine services across all conditions and in the subcohorts of the six most frequent hospital conditions and three pain-associated conditions. This represents an institutional and societal opportunity for quality improvement initiatives to promote equitable pain management.

## 1. Introduction

The opioid epidemic continues to grow, with a record 107,270 drug overdose deaths in 2021, up from 92,478 in 2020 [1]. This has brought national attention to opioid prescribing practices and ways to curb the epidemic. While deprescribing and utilizing multi-modal pain approaches is important, there are concerns that certain marginalized patient groups may receive disproportionately fewer opioid medications and inadequate pain control and may more likely develop chronic pain [2, 3]. Research on opioid prescribing practices in the hospital setting can help to address undertreated pain, overprescribing, overdose, and deaths.

Multiple studies to date have identified notable differences in opioid prescribing practices in emergency medicine care for patients with limited English proficiency (LEP) with traumatic injuries [4] and in children receiving surgical care [5, 6]. There is also a substantial body of literature on racial and ethnic inequities in opioid prescribing in emergency department [7–12] and primary care settings [12, 13]. We previously published a study of inequities in discharge opioid prescriptions from internal medicine services [12], but less is known about inequities in opioid administration for medical inpatients while hospitalized. There is a substantial gap in the literature for how pain is treated and the related opioid requirements for common medical conditions treated in hospitalized patients. Moreover, understanding differences seen in conditions typically associated with pain will provide greater context as we design future quality improvement interventions designed to promote equity. This study attempts to address shortcomings of our prior analysis and focuses on the inpatient administration of opioids. Better clarifying opioid prescribing practices in the inpatient internal medicine setting will allow for the identification of inequities and lead to subsequent efforts to optimize pain management for a diverse group of patients and conditions treated.

The objective of our study was to assess whether race, ethnicity, and language status were associated with opioid prescriptions for patients hospitalized on an internal medicine service across all medical conditions, the most frequent treated conditions, and common pain-associated conditions.

## 2. Methods

### 2.1. Study Design, Setting, Data Sources

We utilized electronic health record (EHR) data from the University of California San Francisco (UCSF) Helen Diller Medical Center, a 785-bed urban academic teaching hospital, to conduct a retrospective cohort study among hospitalized adults discharged from the internal medicine services from January 2013 to September 2021. All data were extracted from our local EHR, Epic, with specific data pulled from Clarity, the relational database that stores Epic data. This included patient demographic and clinical data, including time-stamped opioid medication administration. This study was approved by the UCSF Institutional Review Board with a waiver of informed consent.

### 2.2. Participants

All admissions for patients aged 18 years or older occurring during the study period were included in the overall cohort. The data were inclusive of each unique admission and included cases of recurrent hospitalizations as the primary hospital conditions and circumstances of each admission can be unique. Both patients who received and did not receive opioids were included. Additional subcohorts included patients whose primary hospital conditions were one of the six most frequent primary hospital conditions (pneumonia, sepsis, cellulitis, gastrointestinal bleed, pyelonephritis/urinary tract infection, or other noninfectious respiratory disease) and three pain-associated primary hospital conditions (abdominal pain, acute back pain, or pancreatitis). A patient's primary hospital condition is designated in the EHR by the discharging clinician and linked to an ICD-10 code. This approach of selecting specific, common, hospital conditions and the most common pain-associated conditions was to reduce confounding by medical condition. For the subcohort of pain-associated conditions, abdominal pain and acute back pain have been previously identified in the literature and pancreatitis was selected as a prominent pain condition by the authors [14]. The process of generating the subcohorts for the six most frequent primary hospital conditions and the select pain conditions is outlined in Supplemental Figure 1 with the list of primary hospital conditions and corresponding ICD-10 codes in Supplemental Table 2.

### 2.3. Variables

The primary outcome of interest was the average amount of daily opioids administered to patients, calculated by oral morphine milliequivalents (MMEs), rounded to the nearest integer value. MMEs are used to compare equivalent dosages across different opioid medications and create a standardized metric for analyses.

We had two primary predictors. The first predictor was limited English proficiency status, defined as patients with a primary language other than English who also required an interpreter. The second predictor was race/ethnicity. This was categorized at time of admission based on patients' self-assignment to one or more racial categories per the U.S. Census Bureau and National Institutes of Health reporting standards (white, black or African American, American Indian or Alaska Native, Asian, and Native Hawaiian or Other Pacific Islander), with additional categories of “multi-race/ethnicity” and “other/unknown.” Patients were classified as ethnically Hispanic/Latinx if Hispanic was their self-reported identity, irrespective of their racial designation. Race/ethnicity categorizations reflect socially, not genetically, designated groupings and serve as a proxy for racism [15, 16].

Additional patient demographic and hospitalization characteristics variables used in the regression analysis included patient age, gender identity, insurance coverage, history of opioid therapy prior to admission, documented history of substance use disorder, methadone or buprenorphine therapy, medical comorbidities, care in the intensive care unit during admission, comfort care/end of life status, pain service or palliative care consultation, year of study, and primary medical team. Opioid therapy prior to admission was determined from the EHR as any opioid medications on admission medication reconciliation. The EHR did not include the specific indication for opioids prescribed prior to admission, but this variable was included because of its close association with the presence of pain-related conditions and need for inpatient analgesia. Substance use was determined in the EHR as a diagnosis included in the hospital problems across all medical center encounters or billing ICD coding. This was included given the strong association substance use disorders have with concurrent pain and opioid prescriptions. Methadone/buprenorphine therapy variables were collected in our EHR based on prescription from two years preceding to six hours after discharge. Insurance status was categorized as Medicare, Medical, Private, Self-Pay, and Other. Primary team was divided between teaching resident medicine services and attending hospitalist-only services. Medical comorbidity was calculated via the Elixhauser index [17].

### 2.4. Statistical Methods

Analyses were performed using Stata software version 17. Patient demographics, hospitalization characteristics, and mean daily MME were stratified by the six most frequent primary hospital conditions and top three pain-associated diagnoses, as well as for each of the individual diagnoses in the latter two groups. Next, we fit a series of negative binomial regression models to examine the association between race, ethnicity, and LEP status and mean daily MME, while adjusting for the patient demographics, hospitalization factors, opioid therapy prior to admission, substance use history, methadone or buprenorphine therapy, year of study, insurance status, and medical comorbidities. For each regression, we also tested for the interaction between race/ethnicity and LEP status. To accomplish this, we assessed goodness of fit for each model and ultimately included the interaction term in the overall model and the models for the six most frequent conditions as there was a significant improvement in model fit when the interaction term was included. There was no improvement in goodness of fit when the interaction between race/ethnicity and LEP status was included for the select pain condition models. Cluster robust variance by medical record number was applied to address readmissions for the same patients within the study period. The negative binomial regression approach was selected as there was a widely dispersed distribution of MMEs, including many patients who did not receive opioid medications during their admission [18, 19]. For the negative binomial regressions, average marginal effects (AMEs) were calculated, effectively modeling population estimates for average opioids received based on the information from our large, population-level dataset. AMEs also enabled us to convey the results of our regression analysis in clinically meaningful units.

## 3. Results

### 3.1. Unadjusted Results

Our study cohort included 61,831 patient hospitalizations. There were 36,237 (59%) patients who received opioid medications during their hospitalization. We included 13,062 patients in the subcohort of the top six most frequent primary hospital conditions and 1,944 patients in the subcohort of the three pain-associated primary hospital conditions (Table 1). These subcohorts were mutually exclusive from each other, but not from the overall cohort. The overall cohort was 83% English speaking and included 17% of patients with LEP. The overall cohort had 46% patients self-identifying as white, 21% Asian, 15% black/African American, 12% Hispanic/Latinx, 2% multi-race/ethnicity, 1% Native Hawaiian/Other Pacific Islander, 0.5% American Indian/Alaska Native, and 4% as other/unknown. The proportion of patients with LEP among the race/ethnicity groupings was as follows: 5% for white, 2% for American Indian/Alaska Native, 46% for Asian, 1% for black/African American, 28% for Latinx, 7% for multi-race/ethnicity, 28% for Native Hawaiian/Other Pacific Islanders, and 14% for other/unknown patients. There were significant differences in the demographic distributions across the overall cohort and subcohorts. Notably, compared to the overall cohort, patients with one of three selected pain diagnoses had higher rates of prescribed opioids (62.4% vs. 41.3%) and methadone or buprenorphine use (7.9% vs. 4.9%). These patients were also younger (52 vs. 62 median age), more female (64.3% vs. 49.2%), and had lower rates of LEP (10.6% vs. 16.9%) (Table 1).

Across all conditions, patients received an average of 68.8 MMEs per day. Patients from the subcohort of top six most frequent primary hospital conditions received an average of 52.7 MMEs per day, and patients from the subcohort of the three select pain-associated diagnoses received an average of 130.88 MMEs per day (Table 2). There are clear differences across patient groups for the overall cohort, subcohorts, and specific diagnoses. Notably, Asian and Latinx patients received among the fewest opioids compared to other racial/ethnic groups. Patients with LEP received fewer opioids compared to non-LEP patients (Table 2).

### 3.2. Main Results

Each model adjusted for patient demographics, hospitalization factors, opioid therapy prior to admission, substance use history, methadone or buprenorphine therapy, year of study, insurance status, and medical comorbidities. All significance testing was performed at the *p* < 0.05 level.

### 3.3. Overall Cohort Regression Analysis

White patients received an average of 103 daily MMEs (95% CI: 94–112). Asian (59 MMEs, 95% CI: 51–66), Latinx (89 MMEs, 95% CI: 79–100), and multi-racial/ethnic (81 MMEs, 95% CI: 65–97) patients received significantly fewer opioids compared to white patients. American Indian/Alaska Native (227 MMEs, 95% CI: 110–344) patients received significantly more daily average opioids compared to white patients. Patients without LEP received an average of 101 daily MMEs (95% CI: 91–111), while patients with LEP received significantly fewer daily opioids (66 MMEs, 95% CI: 52–80).

### 3.4. Subgroup Regression Analysis: Top Six Most Frequent Hospital Conditions

White patients received an average of 175 daily MMEs (95% CI: 142–207). Asian (83 MMEs, 95% CI: 64–102) and Latinx (137 MMEs, 95% CI: 102–172) patients received significantly fewer opioids compared to white patients. American Indian/Alaska Native patients received significantly more opioids (483 MMEs, 95% CI: 322–644) compared to white patients. Non-LEP patients received an average of 155 daily MMEs (95% CI: 129–182), while patients with LEP received significantly less, an average of 114 daily MMEs (95% CI: 85–144).

### 3.5. Subgroup Regression Analysis: Three Pain-Associated Conditions

White patients received an average of 157 MMEs (95% CI: 138–175). Asian (108 MMEs, 95% CI: 85–130), black (122 MMEs, 95% CI: 100–144), Latinx (126 MMEs, 95% CI: 106–147), Native Hawaiian and Other Pacific Islander (68 MMEs, 95% CI: 35–101), and patients categorized as other (106 MMEs, 95% CI: 80–131) received significantly fewer opioids compared to white patients. Non-LEP patients received an average of 141 daily MMEs (95% CI: 129–154), while patients with LEP received significantly fewer daily opioids (77 MMEs, 95% CI: 58–96).

**Figure 1:**
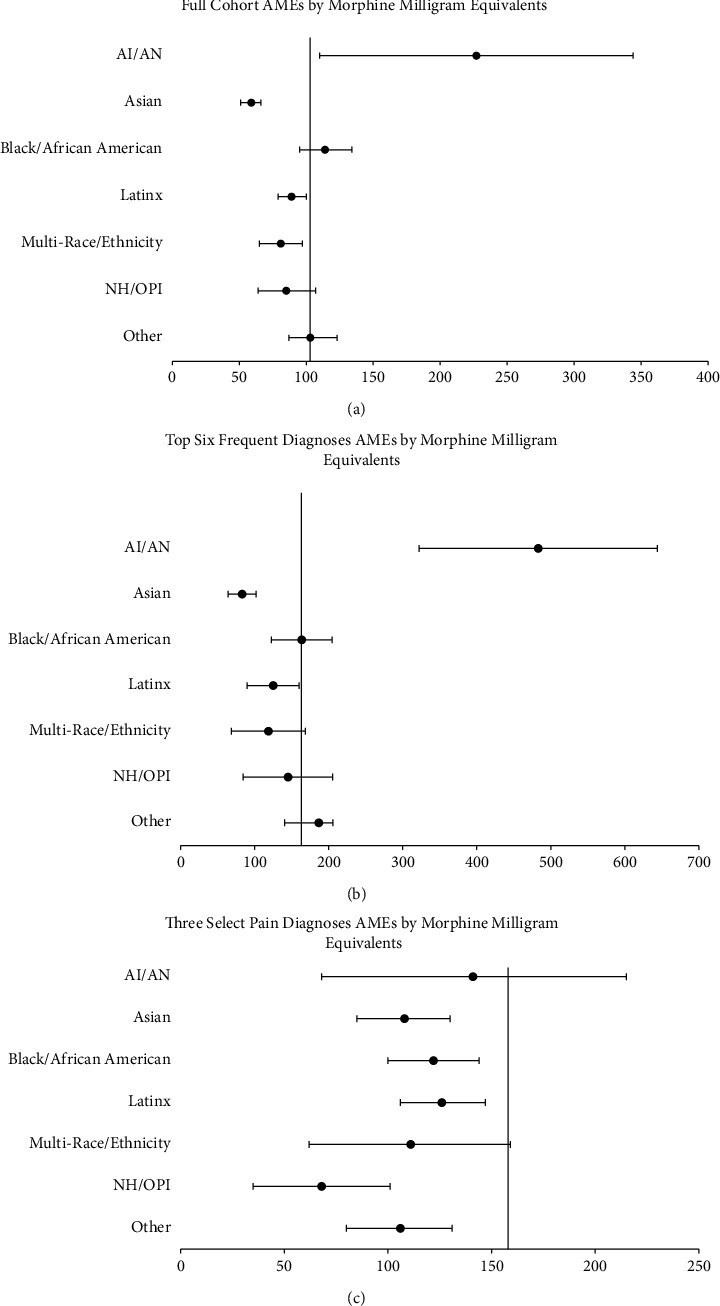
Forest plots of negative binomial regression results by race/ethnicity reported via average marginal effects (AMEs). The baseline comparison for race/ethnicity is white. AI/AN, American Indian or Alaska Native; LEP, limited English proficiency. NH/OPI, Native Hawaiian or Other Pacific Islander.

Forest plots of the average marginal effects across race/ethnicity from the negative binomial regression are available in Figure 1. Negative binomial regression results for the overall cohort, top six most frequent conditions, and three pain-associated conditions are available in Table 3, with results for all covariates utilized in the regression analyses available in Supplemental Table 1.

## 4. Discussion

In this study, we found notable racial, ethnic, and language-based inequities in opioid prescribing for patients hospitalized on internal medicine services. Patients with LEP received significantly fewer opioids in general, when treated for the most frequent conditions and when treated for conditions typically associated with pain such as abdominal pain, acute back pain, and pancreatitis. Asian and Latinx patients received significantly fewer opioids than white patients in the overall cohort and in the subcohorts of the six most frequent primary hospital conditions and three select pain-associated conditions. Notably, black patients did not receive significantly different opioid medications than white patients in the overall cohort but received significantly fewer opioid medications in the subcohort of the select pain-associated conditions.

Our findings suggest that differences in prescribing practices by race, ethnicity, and language persist even after controlling for factors such as intensive care admission, consultation with a pain or palliative care service, comorbidities, opioid use prior to admission, and methadone or buprenorphine therapy. Importantly, our regression models also incorporated interaction terms to accurately reflect the complex relationships that exist between the variables in our models. Patients with LEP often have lower quality and satisfaction of care and understanding of treatment plans and experience a higher incidence of medical errors [20–25]. Language differences may lead to physician biases, and the time required to utilize interpreter services may lead to limited pain assessments and inequitable pain medicine prescriptions [4, 26, 27]. Racial and ethnic inequities in opioid prescribing may be driven by clinician bias in pain assessment and institutional and structural racism [28].

These findings build on our previous research demonstrating racial and ethnic inequities in discharge opioid prescribing across all conditions on internal medicine services [13]. We have now identified that opioid prescribing inequities exist for inpatient treatment in addition to discharge prescriptions we previously studied. It is challenging to identify ways to promote more equitable prescribing across all conditions given the extreme heterogeneity in clinical circumstances encountered in internal medicine. In this current study, our stepwise approach of evaluating all conditions together (*n* = 61,831), top 6 conditions (*n* = 13,062), and 3 select pain conditions (*n* = 1,944) allows for a more nuanced understanding of where inequities are most prevalent in the inpatient setting. This granular approach can help to identify areas for practice change and quality improvement. For example, we found that black patients did not receive significantly different opioids across all conditions in the overall cohort, but for the top three pain-related conditions, they received substantially fewer compared to white patients. We found that patients with LEP received fewer opioids compared to non-LEP patients in the overall cohort, for the top 6 conditions and the three select pain conditions. Using pancreatitis as a case example, black patients received nearly 40% fewer opioids for a pancreatitis admission compared to white patients. Patients with LEP received an average of 30.8 MMEs/day while English-speaking patients received 136.2 MMEs/day, a more than fourfold difference. These are clinically meaningful differences. A standard tablet of 5 mg oxycodone is equivalent to 7.5 MMEs. Patients with LEP and pancreatitis in the above example, on average, would receive the equivalent of 14 fewer 5 mg tablets of oxycodone per day. Collecting these data at an institutional or departmental level enables clinicians to identify tangible targets for improvement to promote equity. Furthermore, focusing on specific diagnoses or groupings of diagnoses that have the most pronounced inequities can enable the formulation of more equitable guidelines for pain management and quality improvement measures for institutions.

Finally, there is an argument that fewer opioids for certain patient groups may be a positive or protective finding [29]. While we recognize the clear harms from opioid overuse and misuse, minoritized and historically disadvantaged groups are most likely to experience bias in the medical profession [2, 30]. Inadequately treated pain has a variety of negative health consequences including reduced quality of life, impaired physical function, high economic costs, and severe psychological and social consequences [3, 31]. The inequitable prescribing practices identified in the pain-associated conditions highlights that further work must be done to eliminate pain management inequities in clinical situations where opioids are often used.

### 4.1. Limitations

There are limitations to our study. First, our data were collected from a single center. While our medical center is diverse, we recognize that practice patterns differ across the nation and some institutions may have pain pathways that promote more standardized care. We do however have nine years of data, with a substantial number of patients, which drives the power behind our findings. Second, our dataset did not allow us to adjust for the reported pain scores and administration of other nonopioid pain medications like acetaminophen and NSAIDs, which can impact opioid medicine prescriptions in the inpatient setting. Third, as we identified the primary hospital conditions for the subgroups, there is a possibility of misclassification in the assignment of primary hospital condition. While our analysis adjusted for comorbidities, opioid, methadone, and buprenorphine use on admission, we are unable to determine the specific indication for each individual opioid administration from the EHR. For example, while a patient may be admitted for cellulitis, a one-time administration of hydromorphone for back pain would not be discernible from our EHR. Non-LEP patients in our cohort received more daily opioids associated with a primary hospital condition of pneumonia than patients with LEP did for abdominal pain, but it is unclear what specific diagnosis or underlying pathophysiology is driving that difference. While our approach of looking at progressively smaller subcohorts is an improvement on previous study designs, there can still be even more granular ways of assessing opioids by indication for specific conditions and even individual clinicians. Fourth, while the regression analyses adjust for comorbidities, our model inherently cannot account for the sum total of hospital problems a patient is being treated for and the complexity of pain that can result from multiple conditions. Fifth, we do not have access to interpreter use data. It would be helpful to know whether the actual use of an interpreter was associated with different medication management for patients with LEP or if there was another mechanism for this inequity.

## 5. Conclusion/Next Steps

Our study demonstrates that racial, ethnic, and language-based inequities in opioid prescribing practices, previously identified in subspeciality and outpatient settings, and on discharge, are also prevalent for patients while hospitalized. To our knowledge, this is the first study to examine opioid prescriptions in the inpatient setting for medicine patients across multiple common medical- and pain-related diagnoses. This contributes to a better understanding of how opioid medications are prescribed for hospitalized patients and how prescribing practices may be optimized to effectively manage pain while minimizing overprescribing and inequitable prescriptions.

Our findings offer directions for future research and quality improvement work to further understand and better address clear inequities in patient care. Within our institution, these findings represent an opportunity to explore the etiology of these inequities and the impact of implicit bias. Our next steps will include a qualitative examination of patients and providers to better understand differences and identify root causes of inequities in pain assessment and management. The identification of specific patient groups (i.e., Asian patients and those with LEP) and specific diagnoses (i.e., pancreatitis) can serve as a starting point for the development of pain management guidelines, like postsurgical care plans in other fields. Expanding work to include analyses beyond our institution will be important to validate these findings across different practice settings and patient populations. Finally, examining the relationship between administered opioid prescriptions and documented pain assessments by nurses and clinicians, incorporating linear mixed and cross-classified multi-level modeling, and differences in scheduled vs. as needed opioids would help to better elucidate causal pathways. In this future approach, we will also explore the possibility of predictive machine learning analyses.

## Figures and Tables

**Table 1:** Baseline patient demographic and clinical characteristics from 2013 to 2021, *n* (%).

Variable	Full cohort (*n* = 61,831)	Top 6 frequent diagnoses (*n* = 13,062)	Top 3 pain diagnoses (*n* = 1,944)
Age, median (IQR)	62 (47–75)	67 (53–80)	52 (37–63)
Male	31,410 (50.8)	6,952 (53.22)	695 (35.75)
English speaking	49,481 (83.12)	9,821 (79.17)	1,708 (89.42)
Limited English proficiency	10,046 (16.88)	2,584 (20.83)	202 (10.58)
Race/ethnicity			
AI/AN	294 (0.48)	56 (0.43)	17 (0.87)
Asian	12,936 (20.92)	3,273 (25.06)	267 (13.73)
Black/African American	9,018 (14.58)	1,661 (12.72)	297 (15.28)
Latinx	7,262 (11.74)	1,430 (10.95)	316 (16.25)
Multi-race/ethnicity	1,303 (2.11)	263 (2.01)	38 (1.95)
NH/OPI	666 (1.08)	201 (1.54)	10 (0.51)
Other/unknown	2,172 (3.51)	430 (3.29)	68 (3.50)
White	28,180 (45.58)	5,748 (44.01)	931 (47.89)
Insurance status			
Medicare	32,461 (52.50)	7,983 (61.12)	716 (36.83)
Medicaid, other indigent	15,614 (25.25)	2,838 (21.73)	669 (34.41)
Private	12,951 (20.95)	2,113 (16.18)	519 (26.70)
Self-pay	502 (0.81)	84 (0.64)	29 (1.49)
Other	303 (0.49)	44 (0.34)	11 (0.57)
Primary team			
Teaching	43,801 (71.21)	9,272 (71.22)	1,320 (68.18)
Hospitalist	17,708 (28.79)	3,747 (28.78)	616 (31.82)
Intensive care management	9,332 (15.09)	2,699 (20.66)	63 (3.24)
Comfort care	2,652 (4.29)	612 (4.69)	27 (1.39)
Pain/palliative consult	5,391 (8.72)	768 (5.88)	247 (12.71)
Opioids on admission	25,521 (41.28)	4,761 (36.45)	1,213 (62.40)
Methadone/buprenorphine	3,013 (4.87)	493 (3.77)	155 (7.97)
Substance use disorder documentation	5,955 (9.63)	915 (7.01)	206 (10.60)
Elixhauser comorbidity score, median (IQR)	8 (0–16)	9 (1–17)	3 (−1–11)
Mean daily opioids (MME)	68.78	52.72	130.88

All comparisons between the subcohorts using chi-squared and ANOVA tests were significant at the *p* < 0.05 level. All values represent no. (%) unless otherwise noted. AI/AN, American Indian or Alaska Native; IQR, interquartile range; MME, oral morphine milligram equivalent; NH/OPI, Native Hawaiian or Other Pacific Islander.

**Table 2:** Mean daily opioid oral morphine milligram equivalents (with standard deviations) by language and race/ethnicity—observed raw data.

Covariate	Full cohort	Top 6 frequent diagnoses	Pneumonia	Sepsis	Cellulitis	GI bleed	Pyelo/UTI^*∗*^	Respiratory disease	3 pain related diagnoses	Abdominal pain	Acute back pain	Pancreatitis
Overall	68.78 (454.77)	52.72 (180.62)	41.34 (158.39)	59.13 (205.23)	86.07 (169.93)	31.20 (143.26)	31.77 (95.09)	79.21 (253.14)	130.88 (202.82)	125.17 (182.23)	173.39 (355.93)	127.77 (172.48)

*Language*												
Non-limited English proficiency	77.40 (494.21)	59.71 (192.42)	47.82 (166.30)	68.79 (225.54)	91.52 (174.21)	34.77 (154.60)	38.51 (104.57)	84.38 (266.48)	141.45 (210.34)	135.80 (189.06)	194.75 (379.30)	136.23 (176.95)
Limited English proficiency	24.31 (107.35)	24.39 (117.39)	21.86 (130.02)	23.08 (88.56)	14.77 (60.91)	16.18 (77.90)	5.39 (27.45)	56.92 (183.47)	39.71 (71.33)	41.95 (75.18)	49.79 (101.51)	30.76 (40.65)

*Race/ethnicity*												
AI/AN	94.34 (242.44)	150.83 (409.29)	161.58 (274.02)	369.35 (975.32)	291.20 (312.95)	26.42 (34.02)	34.79 (39.25)	87.86 (166.28)	173.92 (102.32)	131.85 (74.07)	194.81 (275.50)	216.03 (65.53)
Asian	27.72 (133.59)	24.15 (120.03)	15.19 (103.60)	23.90 (101.79)	21.88 (52.24)	17.97 (89.47)	11.41 (61.14)	56.99 (206.99)	67.17 (135.52)	83.52 (164.08)	41.15 (95.60)	49.41 (81.21)
Black/African American	119.87 (425.49)	64.10 (161.36)	66.51 (151.78)	83.15 (195.56)	72.82 (148.79)	33.47 (77.78)	52.99 (135.12)	68.88 (200.18)	125.49 (165.17)	129.31 (147.50)	195.96 (333.26)	98.27 (101.90)
Latinx	63.23 (214.02)	60.63 (207.26)	58.57 (234.08)	61.37 (197.02)	72.26 (158.40)	43.89 (149.93)	38.11 (81.26)	96.49 (307.89)	121.88 (159.62)	139.30 (170.40)	152.06 (190.03)	100.94 (127.05)
Multi-race/ethnicity	59.85 (551.98)	31.34 (81.48)	34.71 (96.07)	25.16 (61.69)	43.84 (76.14)	11.87 (21.99)	38.88 (88.87)	50.49 (121.21)	94.39 (111.88)	134.70 (150.14)	99.87 (93.95)	61.42 (64.95)
NH/OPI	80.30 (978.62)	50.08 (130.96)	29.77 (72.73)	70.91 (176.18)	73.11 (165.94)	27.89 (72.11)	16.12 (21.51)	120.40 (221.89)	67.17 (111.74)	101.33 (136.98)	10.56 (5.66)	21.30 (30.13)
Other/unknown	77.68 (276.84)	87.95 (312.91)	66.58 (289.33)	85.28 (232.99)	77.94 (159.23)	41.24 (97.13)	12.95 (26.43)	204.57 (569.95)	124.38 (160.25)	124.73 (132.44)	213.08 (304.55)	83.78 (104.66)
White	71.88 (575.34)	61.23 (192.69)	43.96 (149.35)	72.16 (243.95)	102.73 (186.13)	38.30 (192.37)	34.81 (100.77)	81.14 (241.54)	155.79 (242.42)	135.77 (208.89)	224.76 (457.98)	162.57 (205.58)

AI/AN, American Indian or Alaska Native; GI, gastrointestinal; LEP, limited English proficiency; NH/OPI, Native Hawaiian or Other Pacific Islander; pyelo, pyelonephritis; UTI, urinary tract infection. ^*∗*^is for the footnote denoting abbreviations for pyelo and UTI.

**Table 3:** Negative binomial regression analysis of daily opioid administration with results for language and race/ethnicity variables.

Covariate	Full cohort; average predicted daily opioid MME (95% confidence interval)	*p* value	Top six frequent conditions; average predicted daily opioid MME (95% confidence interval)	*p* value	Three pain conditions; average predicted daily opioid MME (95% confidence interval)	*p* value
*Language*						
Non-limited English proficiency	101 (91–111)	Ref	155 (129–182)	Ref	141 (129–154)	Ref
Limited English proficiency	66 (52–80)	<0.001	114 (85–144)	0.002	77 (58–96)	<0.001

*Race/ethnicity*						
White	103 (94–112)	Ref	175 (142–207)	Ref	157 (138–175)	Ref
American Indian/Alaska Native	227 (110–344)	0.036	483 (322–644)	<0.001	141 (68–215)	0.687
Asian	59 (51–66)	<0.001	83 (64–102)	<0.001	108 (85–130)	<0.001
Black/African American	114 (95–134)	0.138	178 (137–219)	0.811	122 (100–144)	0.010
Latinx	89 (79–100)	0.006	137 (102–172)	0.020	126 (106–147)	0.033
Multi-race/ethnicity	81 (65–97)	0.005	129 (79–179)	0.059	111 (62–159)	0.075
Native Hawaiian/Other Pacific Islander	85 (64–107)	0.091	149 (88–209)	0.387	68 (35–101)	<0.001
Other/unknown	105 (87–123)	0.798	188 (142–207)	0.628	106 (80–131)	0.001

MME, oral morphine milligram equivalent. Predicted population rates using average marginal effects. The models utilized complete case regression. For the regressions including the full cohort and the top 6 frequent conditions, the interaction term between race/ethnicity and LEP status was included because the likelihood ratio test was significant comparing the models with and without the interaction term. For the regression of top 3 pain conditions, the interaction between race/ethnicity and LEP was not included because the likelihood ratio test was nonsignificant.

## Data Availability

The retrospective cohort data used to support the findings of this study have not been made available because of patient privacy.

## Supplementary Materials

Supplementary Materials Supplemental Table 1: negative binomial covariate regression results with average marginal effects. Table with full cohort, top six frequent conditions, and three select pain conditions and corresponding negative binomial incidence rate ratios, 95% confidence interval, and *p* values for all covariates. Supplemental Figure 1: selection of the six most frequent and three pain-associated diagnoses. Flow diagram depicting hospitalizations in study period, assigned primary hospital condition, and author process of selecting top six frequent conditions and three select pain-associated conditions. Supplemental Table 2: primary hospital conditions, ICD-10 codes. This table includes all of the ICD-10 codes and their associated sample sizes that were combined to classify the diagnoses used in this study.
