# Supplementary material for: Racial, Ethnic, and Language-Based Inequities in Inpatient Opioid Prescribing by Diagnosis from Internal Medicine Services, a Retrospective Cohort Study
Source: Pain Res Manag. 2023 Sep 21;2023:1658413. doi: 10.1155/2023/1658413 (PMC10539084; doi:10.1155/2023/1658413)
Supplement: Supplementary Materials — Supplemental Table 1: negative binomial covariate regression results with average marginal effects. Table with full cohort, top six frequent conditions, and three select pain conditions and corresponding negative binomial incidence rate ratios, 95% confidence interval, and p values for all covariates. Supplemental Figure 1: selection of the six most frequent and three pain-associated diagnoses. Flow diagram depicting hospitalizations in study period, assigned primary hospital condition, and author process of selecting top six frequent conditions and three select pain-associated conditions. Supplemental Table 2: primary hospital conditions, ICD-10 codes. This table includes all of the ICD-10 codes and their associated sample sizes that were combined to classify the diagnoses used in this study. [file 1658413.f1.docx]

Racial, ethnic, and language-based inequities in inpatients opioid prescribing by diagnosis from internal medicine services

**Supplementary Materials**

**Supplemental Table 1**. Negative binomial covariate regression results with average marginal effects

| **Covariate** | Full Cohort;  Average Predicted Daily Opioid MME (95% Confidence Interval) | p-value | Top Six Frequent Conditions;  Average Predicted Daily Opioid MME (95% Confidence Interval) | p-value | Three Pain Conditions; Average Predicted Daily Opioid MME (95% Confidence Interval) | p-value |
| --- | --- | --- | --- | --- | --- | --- |
| **Gender** |  |  |  |  |  |  |
| Female | 95 (86-104) | ref | 136 (111-161) | ref | 134 (121-148) | ref |
| Male | 103 (92-115) | 0.014 | 168 (139-197) | <0.001 | 145 (128-163) | 0.243 |
| **Primary Team** |  |  |  |  |  |  |
| Hospitalist Only | 109 (96-121) | ref | 169 (135-202) | ref | 150 (131-169) | ref |
| Med Teaching Team | 97 (87-106) | <0.001 | 152 (126-179) | 0.097 | 132 (120-144) | 0.039 |
| **ICU Admission** |  |  |  |  |  |  |
| No | 66 (60-73) | ref | 49 (43-54) | ref | 134 (122-146) | ref |
| Yes | 265 (231-300) | <0.001 | 412 (341-483) | <0.001 | 250 (146-354) | 0.029 |
| **Opioids on Admission** |  |  |  |  |  |  |
| No | 33 (30-35) | ref | 46 (39-53) | ref | 59 (51-67) | ref |
| Yes | 152 (139-166) | <0.001 | 252 (210-293) | <0.001 | 171 (156-185) | <0.001 |
| **Pain/Palliative Care Consult** |  |  |  |  |  |  |
| No | 74 (66-81) | ref | 113 (96-131) | ref | 116 (106-126) | ref |
| Yes | 181 (163-199) | <0.001 | 277 (222-333) | <0.001 | 223 (187-260) | <0.001 |
| **Substance Use History** |  |  |  |  |  |  |
| No | 99 (89-109) | ref | 151 (125-177) | ref | 137 (125-150) | ref |
| Yes | 101 (89-113) | 0.610 | 172 (133-211) | 0.178 | 144 (114-174) | 0.660 |
| **MOUD** |  |  |  |  |  |  |
| No | 74 (70-79) | ref | 117 (98-135) | ref | 123 (113-134) | ref |
| Yes | 260 (206-313) | <0.001 | 396 (302-490) | <0.001 | 218 (169-267) | <0.001 |
| **Comfort Care** |  |  |  |  |  |  |
| No | 81 (72-89) | ref | 96 (84-109) | ref | 136 (124-148) | ref |
| Yes | 367 (307-427) | <0.001 | 750 (538-962) | <0.001 | 220 (60-379) | 0.308 |
| **Insurance** |  |  |  |  |  |  |
| Medicare | 86 (77-95) | ref | 128 (103-153) | ref | 142 (121-162) | ref |
| Medi-cal (Medicaid in California) | 125 (110-140) | <0.001 | 209 (169-248) | <0.001 | 143 (126-160) | 0.908 |
| Private | 79 (71-87) | 0.070 | 114 (91-138) | 0.195 | 127 (111-143) | 0.184 |
| Self-Pay | 156 (91-222) | 0.037 | 111 (53-169) | 0.564 | 139 (74-204) | 0.932 |
| Other | 79 (53-105) | 0.593 | 100 (20-180) | 0.491 | 102 (38-165) | 0.243 |
| **Other** |  |  |  |  |  |  |
| Age | -3 (-4- -3) per year | <0.001 | -7 (-9- -6) per year | <0.001 | -3 (-3- -2) per year | <0.001 |
| Time | -7 (-8- -5) per year | <0.001 | -9 (-13- -5) per year | <0.001 | -8 (-12- -5) per year | <0.001 |
| Mortality Score | -1 (-1- -1) per point | <0.001 | -2 (-2- -1) per point | <0.001 | -2 (-3- -1) per point | <0.001 |

Abbreviations: ICU, Intensive Care Unit; MOUD, Medications for Opioid Use Disorder (Methadone, Buprenorphine)

**Supplemental Figure 1.** Selection of six most frequent and three pain-associated diagnoses.

61,831 adult hospitalizations on internal medicine services

January 2013-September 2021

8,663 unique primary hospital conditions assigned

1,472 primary hospital conditions with ≥5 hospitalizations

7,191 primary hospital conditions with <5 hospitalizations

Six most frequent primary hospital conditions selected, combining like terms*

Three pain-associated primary hospital conditions selected, combining like terms*

Pneumonia

Sepsis

Cellulitis

Gastrointestinal Bleed

Pyelonephritis; Urinary Tract Infection

Respiratory Disease

Abdominal Pain

Acute Back Pain

Pancreatitis

*Like terms combined as per supplemental table 2

**Supplemental Table 2.** Primary hospital conditions, ICD-10 Codes.

| Six Most Frequent Primary Hospital Conditions | Combined Primary Hospital Conditions (Count) | ICD-10 Code | Total Sample Size |
| --- | --- | --- | --- |
| Pneumonia | Pneumonia (1,355)  Covid-19 (321)  CAP (community acquired pneumonia) (297)  Aspiration pneumonia (296)  Community acquired pneumonia (240)  HCAP (healthcare-associated pneumonia) (219)  Pneumonia, unspecified organism (123)  Pneumonia due to COVID-19 virus (102)  PNA (pneumonia) (73)  COVID-19 virus infection (52)  Healthcare-associated pneumonia (28)  Multifocal pneumonia (27)  Community acquired bacterial pneumonia (25)  Viral pneumonia (19)  Necrotizing pneumonia (16)  Recurrent aspiration pneumonia (16)  Atypical pneumonia (15)  Hospital-acquired pneumonia (14)  Organizing pneumonia (14)  RSV (respiratory syncytial virus pneumonia) (13)  Acute respiratory distress syndrome (ARDS) due to COVID-19 virus (10)  Postobstructive pneumonia (10)  Acute respiratory disease due to COVID-19 virus (7)  HAP (hospital-acquired pneumonia) (7)  Influenza A with pneumonia (7)  MRSA pneumonia (7)  Pneumonia, community acquired (7)  Recurrent pneumonia (6)  COVID-19 virus detected (5)  Fungal pneumonia (5)  History of 2019 novel coronavirus disease (Covid-19) (5)  History of COVID-19  Influenza, pneumonia (5)  PCP (pneumocystis jiroveci pneumonia) (5)  Pneumonia and influenza (5) | J18.9  U07.1  J18.9  J69.0  J18.9  J18.9  J18.9  U07.1, J12.82  J18.9  U07.1  J18.9  J18.9  J15.9  J12.9  J85.0  J69.0  J18.9  J18.9, Y95  J84.89  J12.1  U07.1, J80  J18.9  U07.1, J06.9  J18.9, Y95  J09.X1  J15.212  J18.9  J18.9  U07.1  B49,J17  Z86.16  Z86.16  IMO0001  B59  J11.00 | 3,361 |
| Sepsis | Sepsis (1,147)  Septic shock (194)  Severe sepsis (173)  Sepsis due to urinary tract infection (157)  Sepsis secondary to UTI (63)  Sepsis due to pneumonia (59)  Sepsis, unspecified (53)  Urosepsis (48)  Sepsis due to undetermined organism (27)  Severe sepsis with acute organ dysfunction (27)  Severe sepsis(995.92) (23)  Severe sepsis with septic shock (22)  Sepsis due to cellulitis (19)  Sepsis due to Escherichia coli (18)  Staphylococcus aureus bacteremia with sepsis (18)  Septic arthritis (17)  Biliary sepsis (13)  Sepsis due to gram negative bacteria (13)  Septic shock due to urinary tract infection (13)  Septic shock(785.52) (13)  Severe sepsis without septic shock (13)  Sepsis due to Escherichia coli (E. coli) (12)  Septic thrombophlebitis (12)  Sepsis associated hypotension (10)  Aseptic meningitis  Septic shock due to Escherichia Coli (8)  MSSA (methicillin susceptible Staphylococcus aureus) septicemia (7)  Sepsis due to gram-negative urinary tract infection (7)  Sepsis due to Staphylococcus aureus (7)  Recurrent sepsis due to urinary tract infection (6)  Sepsis affecting skin (6)  Sepsis due to gram-negative URI (5)  Sepsis due to Klebsiella pneumoniae (5)  Sepsis due to undetermined organism, with acute renal failure (5)  Septic arthritis of hip (5)  Septic arthritis of knee, left (5) | A41.9  A41.9, R65.21  A41.9, R65.20  A41.9, N39.0  A41.9, N39.0  J18.9, A41.9  N/A  A41.9, N39.0  A41.9  A41.9, R65.20  N/A  A41.9, R65.21  L03.90, A41.9  A41.51  A41.01  M00.9  K83.09  A41.50  A41.9, R65.21, N39.0  N/A  A41.9, R65.20  A41.51  I80.9  A41.9, I95.9  G03.0  A41.51, R65.21  A41.01  A41.50, N39.0  A41.01  A41.9, N39.0  A41.9  A41.50, N39.0  A41.4  A41.9, R65.20, N17.9  M00.9  M00.9 | 2,239 |
| Cellulitis | Cellulitis (992)  Cellulitis and abscess of hand (26)  Cellulitis and abscess of leg (26)  Cellulitis and abscess (24)  Cellulitis of hand (24)  Cellulitis of right leg (23)  Cellulitis of right lower extremity (21)  Cellulitis of left leg (19)  Cellulitis of leg, right (18)  Left leg cellulitis (18)  Preseptal cellulitis (16)  Cellulitis of left lower extremity (15)  Facial cellulitis (14)  Abscess and cellulitis (13)  Cellulitis and abscess of foot (12)  Preseptal cellulitis of right eye (12)  Preseptal cellulitis of left eye (11)  Recurrent cellulitis of lower leg (11)  Cellulitis and abscess of trunk (10)  Cellulitis and abscess of right lower extremity (9)  Cellulitis of left hand (9)  Cellulitis of leg, left (9)  Cellulitis of arm, left (8)  Orbital cellulitis on left (8)  Cellulitis and abscess of right leg (7)  Cellulitis of right hand (7)  Cellulitis and abscess of left leg (6)  Cellulitis of hand, right (6)  Cellulitis of left lower leg (6)  Cellulitis of left upper extremity (6)  Cellulitis of leg (6)  Cellulitis and abscess of buttock (5)  Cellulitis of arm, right (5)  Cellulitis of foot (5)  Cellulitis of right lower leg (5)  Orbital cellulitis (5)  Right arm cellulitis (5) | L03.90  L03.119, L02.519  L03.119, L02.419  L03.90, L02.91  L03.119  L03.115  L03.115  L03.116  L03.115  L03.116  L03.213  L03.116  L03.211  L03.90, L02.91  L03.119, L02.619  L03.213  L03.213  L03.119  L03.319, L02.219  L03.115, L02.415  L03.114  L03.116  L03.114  H05.012  L03.115, L02.415  L03.113  L03.116, L02.416  L03.113  L03.116  L03.114  L03.119  L02.21, L03.317  L03.113  L03.119  L03.115  H05.019  L03.113 | 1,422 |
| Gastrointestinal Bleed | GI bleed (465)  Upper GI bleed (292)  Hemoptysis (207)  GIB (gastrointestinal bleeding) (204)  Gastrointestinal hemorrhage (174)  UGIB (upper gastrointestinal bleed) (156)  Lower GI bleed (137)  Melena (137)  Hematochezia (124)  Acute upper GI bleed (42)  GI bleeding (34)  Gastrointestinal hemorrhage with melena (26)  Upper GI bleeding (24)  Rectal bleeding (23)  Acute lower GI bleeding (22)  Acute GI bleeding (20)  Gastrointestinal bleed (14)  Lower GI bleeding (12)  Cough with hemoptysis (11)  Gastrointestinal bleeding (10)  UGI bleed (10)  Gastrointestinal hemorrhage with hematemesis (8)  Massive hemoptysis (8)  Ulcerative colitis with rectal bleeding (8)  Esophageal varices with bleeding (7)  GI bleed due to NSAIDs (7)  History of GI bleed (7)  Acute ulcerative colitis with rectal bleeding (6)  Acute upper GI bleeding (6)  Anemia due to GI blood loss (6)  GI (gastrointestinal bleed) (5)  GI bleed requiring more than 4 units of blood in 24 hours, ICU, or surgery (5)  Lower gastrointestinal bleed (5)  Upper gastrointestinal bleed (5) | K92.2  K92.2  R04.2  K92.2  K92.2  K92.2  K92.2  K92.1  K92.1  K92.2  K92.2  K92.1  K92.2  K62.5  K92.2  K92.2  K92.2  K92.2  R04.2  K92.2  K92.2  K92.0  R04.2  K51.911  I85.01  K92.2, T39.395A  Z87.19  K51.911  K92.2  D50.0  K92.2  K92.2  K92.2  K92.2 | 2,227 |
| Pyelonephritis; Urinary Tract Infection | Pyelonephritis (480)  UTI (urinary tract infection) (412)  Urinary tract infection (224)  UTI (lower urinary tract infection) (141)  Complicated UTI (urinary tract infection) (136)  UTI (urinary tract infection), uncomplicated (38)  Complicated urinary tract infection (33)  Recurrent UTI (33)  Pyelonephritis, acute (22)  Acute pyelonephritis (21)  Pyelonephritis due to Escherichia coli (13)  UTI due to extended-spectrum beta lactamase (ESBL) producing Escherichia coli (13)  UTI (urinary tract infection) due to urinary indwelling catheter (12)  Catheter-associated urinary tract infection (11)  Recurrent UTI (urinary tract infection) (8)  Lower urinary tract infectious disease (6)  E-coli UTI (5)  Recurrent pyelonephritis (5) | N12  N39.0  N39.0  N39.0  N39.0  N39.0  N39.0  N39.0  N10  N10  N12, B96.20  N39.0, B96.29, Z16.12  T83.511A, N39.0  T83.511A, N39.0  N39.0  N39.0  N39.0, B96.20  N12 | 1,613 |
| Respiratory Disease | Hypoxia (361)  Shortness of breath (339)  Dyspnea (229)  Acute hypoxemic respiratory failure (220)  Respiratory failure (215)  SOB (shortness of breath) (122)  Hypoxemia (99)  Acute respiratory failure with hypoxia and hypercarbia (66)  Respiratory failure with hypercapnia (54)  Respiratory failure with hypoxia (52)  ARDS (acute respiratory distress syndrome (38)  Respiratory failure with hypoxia and hypercapnia (37)  Dyspnea on exertion (36)  Respiratory distress (33)  Acute respiratory failure with hypoxia (28)  Hypoxemic respiratory failure, chronic (28)  Acute respiratory failure (24)  Respiratory failure, acute (20)  Acute respiratory failure with hypoxia (19)  Dyspnea and respiratory abnormalities (17)  Acute respiratory failure with hypoxemia (14)  Acute respiratory failure with hypoxia and hypercapnia (14)  DOE (dyspnea on exertion) (14)  Acute respiratory failure with hypercapnia (13)  RSV (acute bronchiolitis due to respiratory syncytial virus) (13)  Acute respiratory distress syndrome (ARDS) (12)  Acute on chronic respiratory failure with hypoxia (10)  RSV (respiratory syncytial virus infection) (10)  Chronic hypoxemic respiratory failure (9)  Hypercapnic respiratory failure (9)  Acute on chronic respiratory failure with hypercapnia (7)  Acute respiratory failure with hypercapnia (7)  Acute on chronic respiratory failure with hypercapnia (6)  Acute and chronic respiratory failure with hypercapnia (5)  Acute hypercapnic respiratory failure (5)  Hypercapnic respiratory failure, chronic (5)  Respiratory failure requiring intubation (5)  Respiratory failure, acute-on-chronic (5) | R09.02  R06.02  R06.00  J96.01  J96.90  R06.02  R09.02  J96.01, J96.02  J92.92  J96.91  J80  J96.91, J96.92  R06.00  R06.03  J96.01  J96.11  J96.00  J96.00  J96.01  R06.00, R06.89  J96.01  J96.01, J96.02  R06.00  J96.02  J21.0  J80  J96.21  B97.4  J96.11  J96.92  J96.21  J96.02  J96.22  J96.22  J96.02  J96.12  J96.90  J96.20 | 2,200 |
| Three Select Pain Diagnoses |  |  |  |
| Abdominal Pain | Abdominal pain (844)  Chronic abdominal pain (34)  RUQ abdominal pain (16)  Acute abdominal pain (15)  Abdominal pain, acute (13)  Epigastric abdominal pain (13)  Abdominal pain, epigastric (12)  Functional abdominal pain syndrome (12)  RUQ pain (10)  Generalized abdominal pain (9)  Intractable abdominal pain (9)  Abdominal pain, other specified site (7)  Rectal pain (7)  Abdominal pain, acute, epigastric (5) | R10.9  R10.9, G89.29  R10.11  R10.9  R10.9  R10.13  R10.13  R10.9  R10.11  R10.84  R10.9  R10.9  K62.89  R10.13 | 1,006 |
| Acute Back Pain | Back pain (136)  Low back pain (31)  Acute back pain (7)  Lower back pain (6)  Back pain of thoracolumbar region (5)  Intractable back pain (5) | M54.9  M54.50  M54.9  M54.50  M54.50, M54.6  M54.9 | 190 |
| Pancreatitis | Pancreatitis (274)  Chronic pancreatitis (178)  Acute pancreatitis (95)  Pancreatitis, acute (24)  Acute on chronic pancreatitis (20)  Gallstone pancreatitis (20)  Pancreatitis, chronic (19)  Pancreatitis, recurrent (16)  Idiopathic chronic pancreatitis (13)  Other chronic pancreatitis (11)  Necrotizing pancreatitis (10)  Acute gallstone pancreatitis (9)  Recurrent pancreatitis (9)  Acute alcoholic pancreatitis (8)  Pancreatitis, unspecified pancreatitis type (8)  Chronic recurrent pancreatitis (7)  Alcoholic pancreatitis (6)  Pancreatitis, alcoholic, acute (6)  Acute biliary pancreatitis without infection or necrosis (5)  Pancreatitis due to biliary obstruction (5)  Pancreatitis, necrotizing (5) | K85.90  K86.1  K85.90  K85.90  K85.90, K86.1  K85.10  K86.1  K85.90  K86.1  K86.1  K85.91  K85.10  K85.90  K85.20  K85.90  K86.1  K85.20  K85.20  K85.10  K85.90, K83.1  K85.91 | 748 |
